# The Aging Process of Cadmium in Paddy Soils under Intermittent Irrigation with Acid Water: A Short-Term Simulation Experiment

**DOI:** 10.3390/ijerph19063339

**Published:** 2022-03-11

**Authors:** Dongya Han, Lixin Pei, Guanxing Huang, Qinxuan Hou, Meng Zhang, Jiangmin Song, Lin Gan, Heqiu Wu

**Affiliations:** 1 Institute of Hydrogeology and Environmental Geology, Chinese Academy of Geological Sciences, Shijiazhuang 050061, China; handongyaycn@126.com (D.H.); houqinxuan@163.com (Q.H.); meng1230331@163.com (M.Z.); 15612773009@163.com (J.S.); ganlin0322@163.com (L.G.); 2 Hebei and China Geological Survey Key Laboratory of Groundwater Remediation, Shijiazhuang 050061, China; 3 Hebei Province Collaborative Innovation Center for Sustainable Utilization of Water Resources and Optimization of Industrial Structure, Hebei GEO University, Shijiazhuang 050031, China; 4 Haikou Marine Geological Survey Center, China Geological Survey, Haikou 571100, China; 5 Zhejiang Engineering Geophysical Survey and Design Institute Co., Ltd., Hangzhou 310005, China; whq76273572@163.com

**Keywords:** Cd fractionation, paddy soils, redox cycles, aging mechanisms

## Abstract

Cadmium (Cd)-contaminated paddy soils are a big concern. However, the effect of irrigation with acid water on the migration and transformation of Cd and the effect of alternating redox conditions caused by intermittent irrigation on Cd aging processes in different depths of paddy soils are unclear. This study revealed Cd fractionation and aging in a Cd-contaminated paddy soil under four irrigation periods with acid water and four drainage periods, by applying a soil columns experiment and a sequential extraction procedure. The results showed that the dynamic changes of soil pH, oxidation reduction potential (ORP), iron (Fe) oxides and dissolved organic carbon (DOC) throughout the intermittent irrigation affected the transformation of Cd fractions. After 32 days, the proportion of exchangeable Cd (F1) to the total Cd decreased with a reduction of 24.4% and 20.1% at the topsoil and the subsoil, respectively. The labile fractions of Cd decreased, and the more immobilizable fractions of Cd increased in the different depths of soils due to the aging process. Additionally, the redistribution of the Fe and Mn oxide-bound Cd (F3) and organic matter and secondary-sulfide-bound Cd (F4) occurred at different depths of soils during the incubation time. Overall, the bioaccessibility of Cd in the subsoil was higher than that in the topsoil, which was likely due to the leaching and accumulation of soluble Cd in the deep soil. In addition, the aging processes in different depths of soils were divided into three stages, which can be mainly described as the transformation of F1 into F3 and F4.

## 1. Introduction

Cadmium (Cd) is a kind of heavy metal with high toxicity, and therefore, Cd contamination in soil is a big concern [1,2,3,4,5]. Cd in soil can cause a threat to human health through the food chain. It may lead to kidney damage, cancer and other chronic diseases and even cause death when humans are exposed to Cd over long periods [6]. In recent years, with the increase in mining activities, large amounts of Cd enter the paddy soil around mining areas due to the accumulation of Cd-containing slag and the drainage of acid mine, which seriously affects food security and the stability of the agricultural foundation. Therefore, it is necessary to carry out research on Cd migration and transformation in paddy soils around mining areas. It is known to all that the mobility and toxicity of Cd are mainly controlled by its fractions instead of the total amount in soils [7,8,9,10]. When exogenous heavy metals enter soils, the bioavailability and mobility of heavy metals in soil decrease over time, which is called “aging” [11].

To date, several studies have suggested that the transformation of Cd fractions is greatly affected by soil properties [12,13,14,15]. For example, Yu et al. [16] confirmed that the addition of humic acid can effectively reduce the mobility and bioaccessibility of Cd in soil. In contrast, the decrease in pH can increase the availability of Cd in soils [17,18]. It was reported that the mobility of Cd in soil was controlled by pH, which can affect the surface charge of minerals, thus affecting the affinity of Cd for a particular sorption site [19,20]. Therefore, the effect of irrigating paddy fields with acid water on the aging process of Cd cannot be ignored. In addition, the aging mechanisms of Cd in soil under different redox conditions have also received much attention. Compared with some variable valence heavy metals (such as arsenic and chromium), Cd is not sensitive to redox conditions [9]. However, the minerals and organic matter, closely associated with the fractions of Cd, can be affected by the ORP in soil, resulting in the transformation of Cd fractions. For example, the reductive dissolution of iron oxides can lead to the release of Cd [21]. During flooding conditions, sulfate (SO_4_^2−^) is reduced to sulfur ions (S^2−^), which forms the precipitation of CdS, reducing the effective form of Cd in soils [22,23,24].

Paddy soils are open and dynamic systems, the flooding and draining cycles can lead to oscillatory changes in soils properties, especially redox potential (Eh) and pH, due to the concerted electron–proton transfers [9,25,26]. So far, most researchers mainly focused on the effect of soil properties (such as pH) on Cd aging under a single aerobic or anaerobic condition, while the effect of an alternating redox condition on the transformation and availability of Cd has received little attention. So, the impact of the alternation of redox conditions caused by intermittent irrigation with acid water on Cd aging in paddy fields needs further research. 

We proposed the hypotheses that the migration and transformation of Cd fractions are the essences of Cd aging in paddy soils, and its aging processes are influenced by intermittent irrigation with acid water. Therefore, the study aimed to investigate (1) the influence of intermittent irrigation with acid water on the fractionation of Cd in different depths of paddy soils over a short time and (2) the aging mechanisms of Cd in different depths of paddy soils affected by intermittent irrigation with acid water. The results will help to accurately assess Cd pollution risk and make adequate remediation strategies in paddy soils.

## 2. Material and Methods

### 2.1. Soil and Analysis

The Cd-uncontaminated topsoil (0–20 cm) was collected from a paddy field (27°14′39″ N, 113°44′10″ E) which is located upstream of the coal mining area in Youxian city, Hunan province, China. The area has a humid subtropical monsoon climate, with an average annual temperature of 17.8 °C and an annual rainfall of 1410 mm. The soil was air-dried, stones and weeds were removed, and it was ground with a pestle, passed through 20-mesh sieves, then thoroughly mixed. 

The soil pH was measured in a 1:2.5 soil-water suspension using a pH meter (S210, SevenCompact, Shanghai, China). The pH electrode was calibrated before each experiment using a calibration buffer, and the estimated precision was 0.01 pH units. The soil particle size and specific area were determined using a laser particle size analyzer (Mastersizer 2000, Malvern, UK). DOC was measured via the K_2_SO_4_ method [27]. The free Fe oxides were determined using a dithionite citrate system buffered with sodium bicarbonate, and the amorphous Fe oxides were determined using ammonium and oxalate under dark conditions [28]. The aqua regia method and modified Tessier’s sequential extraction procedure were used to measure the values of total Cd and Cd fractions in soils, respectively [29,30]. Then, the water-soluble Cd was determined using a graphite furnace atomic absorption spectrophotometer (PinAAcle900T, Shanghai, China). All solutions were prepared using analytical grade materials. The physicochemical properties of the soils are shown in Table 1.

### 2.2. Experimental Scheme

**Figure 1:**
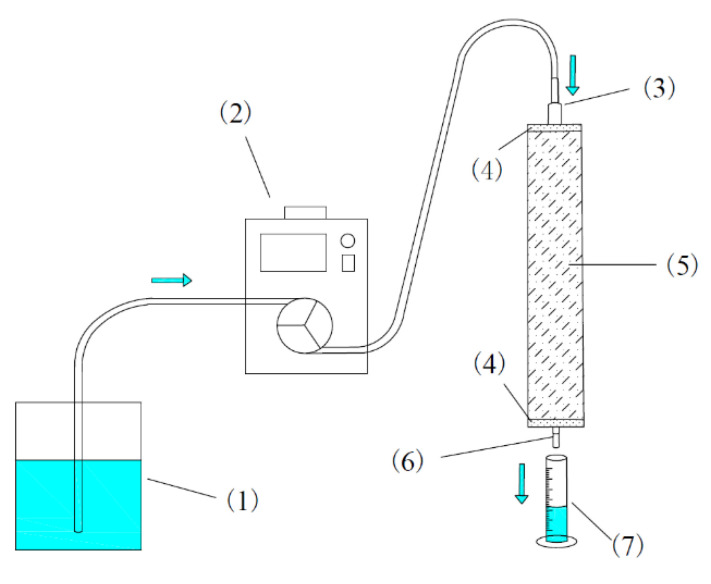
Experimental set-up of the soil column: (1) reservoir, (2) peristaltic pump, (3) inlet, (4) quartz sand, (5) soil, (6) outlet, (7) waste.

Soil columns design: eight organic glass columns, which have a diameter of 5.00 cm and a length of 25.00 cm, with rough interfaces to prevent the effect of dominant flow, were prepared in a room-temperature culture chamber. Additionally, on the ends of the soil columns, two inlet–outlet holes were installed; the top one connected the reservoir through a peristaltic pump, and another was used to drain water. The experiment set-up is shown in Figure 1.

Experiment design: A total of 2.55 kg soil was weighed from the soil samples, and 1.4 L 1.64 × 10^−4^ mol/L Cd (NO_3_)_2_ was added and stirred well; then, the content of exogenous Cd in soil increased by 10 mg/kg. The purpose of this was to make the results more obvious. The treated soils were freeze-dried, ground, and passed through 20-mesh sieves. A small amount of soil was analyzed for the total Cd and fractions of Cd; the others were used to fill the soil columns. Quartz sands (particle size: 70–120 mesh) were soaked in 10% hydrochloric acid and washed with deionized water until the electrical conductivity of supernatant was less than 10 μs/cm. The soil columns were filled with 10 cm of the composite soil, the soil was compacted continuously during the filling process, and 1 cm of quartz sand was added to the top and bottom of the soil columns, and a layer of gauze was placed above and below the quartz sands, respectively. This was performed to avoid soil loss and outlet blockage. 

Intermittent irrigation was simulated by controlling the flow of water through the soil columns in the laboratory. Firstly, a peristaltic pump was used to input the deionized water from the bottom of the columns at a slower rate to remove the air in the soil columns; then, the direction of the flow was adjusted to form a steady flow field. Then, 0.01 mol/L NaCl was used to simulate acidic irrigation water, and the pH of the solution was adjusted to 3.7 ± 0.2. After the flow rate was stable, 0.01 mol/L NaCl was pumped into the soil column at a flow rate of 0.5 mL/min for 4 h, then stopped for 7 d. Moreover, the cover on the soil column was opened to maintain soil column air circulation in the drainage stage. In this way, one day of irrigation and seven days of cyclic ventilation were achieved, that is, eight days made up an intermittent irrigation cycle. The experiment lasted for four cycles. After stopping irrigation (the strongest reduction state) and on the last day before the next cycle of water supply (the strongest oxidation state), the columns were broken at the time that the irrigation stopped and on the last day of the hydrophobic period, respectively. Then, a group of soil samples were taken from the topsoil and subsoil of the columns and denoted as L_T_ and L_S_, respectively. The soil samples were freeze-dried in a vacuum, and subsequently, the physical and chemical properties (including ORP, pH, DOC) and the total Cd and Cd fractions of the samples were analyzed. According to the analysis results, the stabilization processes of Cd in contaminated soil under intermittent irrigation were discussed.

### 2.3. Sequential Extraction Procedure of Cd

A modified Tessier’s sequential extraction procedure (SEP) was applied in the study to analyze the fractions of Cd in the soil (Table 2) [30], where the following details were adjusted: the volume of the extraction solvents and the shaking time of exchangeable Cd (F1), carbonate-bound Cd (F2), Fe and Mn oxide-bound Cd (F3) and organic matter and secondary-sulfide-bound Cd (F4) increased, and the concentration of the extraction solvents of F4 also increased. In addition, to ensure the safety of residual Cd (F5) extraction, instead of the HF-HClO_4_ mixture, aqua regia was applied. The protocol of SEP was as follows: 1 g of dried soil sample was weighed into a 50 mL centrifuge tube and was subsequently extracted sequentially with 40 mL of 1 mol/L magnesium chloride (MgCl_2_) that corresponded to F1; 40 mL of 1 mol/L sodium acetate (NaOAc) that corresponded to F2; 40 mL of 0.25 mol/L hydroxylamine hydrochloride buffer (NH_2_OH·HCl) that corresponded to F3; 24 mL of 30% hydrogen peroxide (H_2_O_2_), 9 mL of HNO_3_, 7.5 mL of 9.6 mol/L ammonium acetate (NH_4_OAc) and 20% HNO_3_ mixture solution that corresponded to F4, which was finally digested by HNO_3_-HClO_4_-HF, which corresponded to F5. All extractions were performed in duplicate, and the extracts were filtered through 0.22 μm filter membranes anterior to Cd determination.

### 2.4. Quality Control and Data Analysis

To guarantee the high quality of the achieved results of the sequential extraction technique, we compared the sum of five Cd fractions with the total Cd; Cd recovery was found to be within the range of 87.2~110.39%. All the data in this study are a mean of the three replicates, and the content of Cd was also calculated with the newly added exogenous Cd (10 mg/kg) after the background value of Cd in the soil was deducted.

## 3. Results and Discussion

### 3.1. Soil Analysis

The properties of the selected soils are summarized in Table 1. In brief, the Cd-uncontaminated soil was composed of 10% clay, 69.16% silt and 19.93% sand, which presented a loamy texture with approximately 0.74 m^2^/g specific surface area. Moreover, the soil was acidic with a pH of 5.47, and the DOC was 25.39 mg/kg. The low pH may be because the acid mine wastewater discharged, and the acidic compounds, which were formed by the reaction of accumulated coal mine and air, entered the soils through rain wash and surface runoff. The contents of the free and amorphous Fe oxides in the study soil were 18.45 mg/kg and 0.51 mg/kg, respectively. The total concentration of Cd in the soil was 0.15 mg/kg, which is lower than the risk screening values (RSVs) of Cd in the soil environmental quality-risk control standard for soil contamination in agricultural land (GB 15618–2018), i.e., 0.30 mg/kg. Obviously, F4 in the soil was the main fraction. 

### 3.2. Soil pH, ORP, Fe and DOC

**Figure 2:**
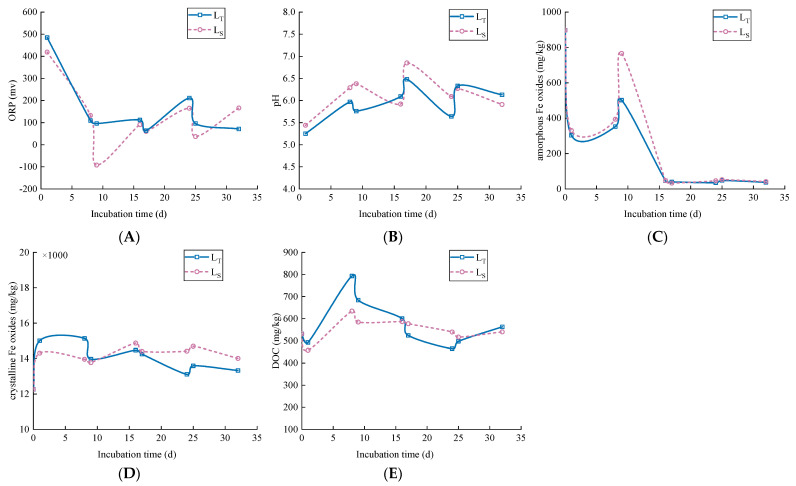
Changes of (**A**) pH, (**B**) ORP, (**C**) amorphous Fe oxides, (**D**) crystalline Fe oxides, (**E**) DOC in the L_T_ and the L_S_ over time under intermittent irrigation.

The soil pH, ORP and the concentration of amorphous Fe oxides, crystalline Fe oxides and DOC, were altered significantly by intermittent irrigation with acid water. The soil properties and components changed in an oscillatory manner, responding to the alternation of soil wetting and drying (Figure 2). 

The intermittent irrigation treatments significantly altered the soil ORP, as the value of ORP decreased with the increase in water (Figure 2A). That is to say, the soil was transformed from a weak oxidation environment to a relative reduction environment under irrigation and from a weak reduction environment to a relative oxidation environment under drying. Furthermore, the ORP of the L_T_ was almost higher than that of the L_S_. The results might be attributed to the deterioration of air penetration into the deeper soil, then a strong O_2_ deficit in the environment under irrigation [31,32,33]. Meanwhile, other researchers have believed that biological and microbiological activities consume oxygen in soil, leading to a decrease in the value of ORP [34]. It is worth mentioning that at the beginning of the intermittent irrigation, the values of ORP displayed a significant decrease, from 484.83 mv to 95.91 mv and from 418.83 mv to −92 mv in L_T_ and L_S_, respectively. 

**Figure 3:**
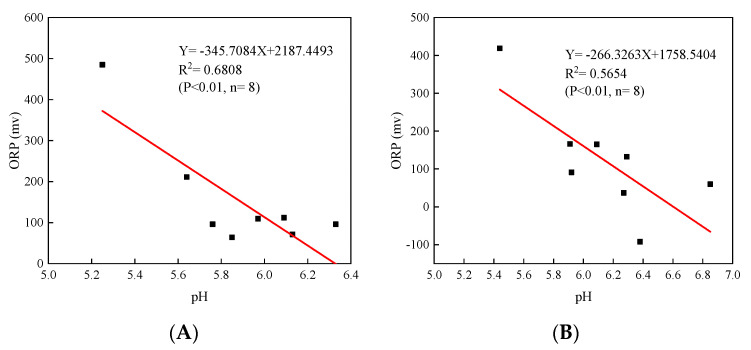
Relationship between soil pH and soil ORP of the (**A**) L_T_ and the (**B**) L_S_.

In this study, the pH in L_T_ and L_S_ presented similar oscillatory changes over time, and the pH in the L_S_ was higher than it was in the L_T_. It is worth noting that when soil is irrigated with acid water, soil pH increases, probably attributed to the combined buffering effect of the irrigation water and soil [35,36]. As shown in Figure 3, the pHs in L_T_ and L_S_ were closely related to the changes in ORP and showed a very significant negative correlation with ORP (*p* < 0.01). Some references have clearly demonstrated the pH decrease is often accompanied by an increase in the ORP in soil solutions [37,38,39]. The increase in pH under reducing conditions in soil might be due to two reasons, i.e., (i) during flooding, organic matter (OM) might be anaerobically degraded through microbial activity, and the protons are consumed, eventually leading to an increase in pH [40]. (ii) H^+^ is consumed as a result of the reduction in oxides, such as NO_3_^−^, SO_4_^2−^ and Fe-oxides under reduction conditions [41,42]. Han et al. [31] attributed the decrease in pH to the oxidative dissolution of FeS in soil during the periods of drainage, and the reaction can be described with Equation (1) [43]. As a whole, the pH remained relatively constant at values between 5.2 and 6.9 throughout the experiments.

(1) $${2\mathrm{FeS}}_{\left(s\right)}+{9/2O}_{2}+{3H}_{2}O=2\mathrm{FeO}(\mathrm{OH})+{2\mathrm{SO}}_{4}^{2-}+{4H}^{+}$$

As is shown in Figure 2C, the content of amorphous Fe oxides in L_T_ and L_S_ presented similar change trends, decreasing rapidly within the first day followed by an increase by 9 d. Then, amorphous Fe oxides decreased sharply and reached the lowest point (about 50 mg/kg) by 16 d in both L_T_ and L_S_, and reached a steady state until the experiment ended. This result indicates that the reciprocal transformation of amorphous and crystalline iron oxides took place within the first 16 d, and iron oxide existed mainly in the crystalline phase in the time that followed. Additionally, the tendency of crystalline Fe oxides to fluctuate was slightly different in the different depths of soils. The content of crystalline Fe oxides increased significantly during the first day of irrigation in both the L_T_ and L_S_, which reached approximately 1.50 × 10^4^ mg/kg and 1.43 × 10^4^ mg/kg, respectively. Then, the content of crystalline Fe oxides decreased in the L_T_ until the experiment ended. By contrast, the content of crystalline Fe oxides in L_S_ rarely changed and remained at 1.40 × 10^4^ mg/kg. In this study, the content of crystalline Fe oxides in the incubation time of 0–9 d was L_T_ > L_S_, but it was L_S_ > L_T_ in the time that followed, indicating that Fe oxides do not crystallize easily in topsoil compared with subsoil. 

As indicated in Figure 2E, during irrigation, DOC in both soils decreased over time; the reason for this might be that OM was degraded by the respiration of anaerobic microbials in the reduction conditions; as mentioned earlier, the process can also cause the increase in soil pH [40,44]. On the contrary, the content of DOC increased during the first drainage period (1–8 d) and the fourth drainage period (25–32 d) in both soils. Notably, there was a difference in the increase rate of DOC during the two periods, where it was significantly higher in the first drainage period than that in the fourth drainage period. That may be because the ORP decreased sharply during the first period, and the soil environment was transformed rapidly from the oxidation to reduction condition so that the OM that bound to Fe/Mn oxides was released by the reductive dissolution of Fe/Mn oxides in soils [39]. 

### 3.3. Influences of Intermittent Irrigation on Cd Fractionation 

**Figure 4:**
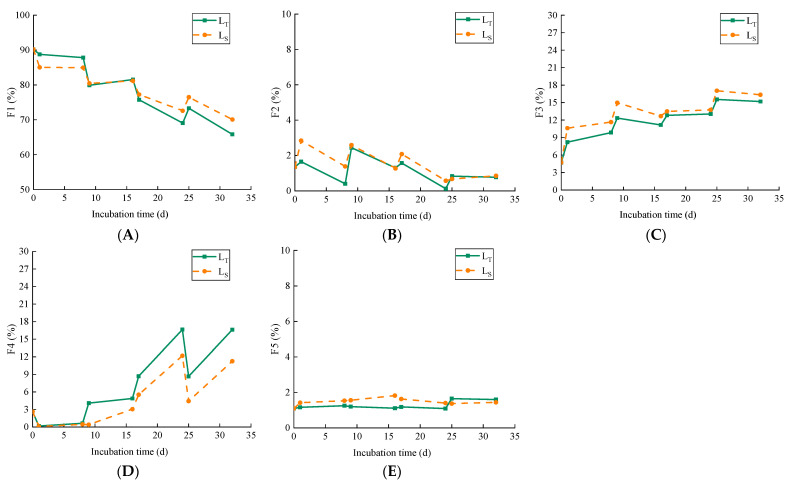
Changes of Cd fractions in the L_T_ and the L_S_ over time under intermittent irrigation ((**A**) exchangeable Cd; (**B**) carbonates bound Cd; (**C**) Fe and Mn oxides bound Cd; (**D**) organic matter and secondary sulfide bound Cd; (**E**) residual Cd).

In order to clearly study the influences of intermittent irrigation on Cd fractionation in soil, changes in the proportion of fractions of Cd in soils over time were observed. As shown in Figure 4, after exogenous Cd was added into soils, the most dominant fraction was F1 at the initial time, reaching about 90%, followed by F3 (about 5%) and F4 (about 3%). The proportions of F2 and F5 were less than 2%. The proportion of F1 was much higher than other fractions, indicating that the addition of exogenous Cd in soils was mainly controlled by the ion exchange at the initial time [30,45]. 

F1 is the most unstable fraction among the five fractions and is most easily taken in and utilized by plants. Several studies have reported that the available Cd had significant positive relationships with water-soluble Cd and exchangeable Cd [46]. In this study, the water-soluble Cd was contained in F1, so, the proportion of F1 can represent the Cd bioaccessibility in soils. As shown in Figure 4A, a similar tendency of F1 was revealed in L_T_ and L_S_ over time; F1 decreased significantly in both L_T_ and L_S_ over time, except the fourth irrigation period (24–25 d). The proportions of F1 decreased to 65.8% and 70.1% with a reduction of 24.4% and 20.1% in the L_T_ and L_S_ after four irrigation–drainage cycles, respectively. The results indicated that F1 was transformed to other fractions in both soils, which was attributed to the effect of aging under intermittent irrigation conditions. However, we found that the proportion of F1 in L_T_ was different from that in L_S_, and the percentage contents of F1 was L_T_ > L_S_ during 0–9 d, while it was greater in L_S_ than L_T_ within the following periods, indicating that the bioaccessibility of Cd increased significantly with increasing depth of the soils. This could be attributed to the fact that the labile Cd desorbed from the upper soil moved down with water flow and accumulated in the bottom layer [47,48]. In general, the proportion of F1 in L_T_ and L_S_ decreased over the incubation time but remained at a high level until the end of the experiment, and it is hypothesized that the decreasing trend will continue. This phenomenon suggested that Cd in paddy soils still showed strong mobility after the incubation time of 32 days [49], and the transformation of labile into more immobilizable fractions in soils may be a long-term process [50].

F2 is a part of Cd that is affiliated to the carbonate precipitate, and it is sensitive to the soil environment. As mentioned earlier, the content of F2 in both L_T_ and L_S_ maintained a low level (<3%) throughout the whole incubation period because of the low content of carbonate in soils. The variation of F2 is shown in Figure 4B. F2 in both L_T_ and L_S_ showed similar change trends, all of which decreased slightly in a fluctuating way. However, the proportion of F2 in L_T_ was slightly higher than in L_S_ in the incubation periods. Several studies have proved that carbonate-bound Cd is susceptible to changes in pH and positively correlated to pH [2,30]. Therefore, the increase in soil pH is beneficial to the formation of carbonate precipitation, while the dissolution of the carbonate precipitation can be dissolved with the decrease in pH, which increased the solubility and bioavailability of Cd in soils [2,51,52]. 

As shown in Figure 4C, F3 in both L_T_ and L_S_ presented a similar change over time, which was characterized by increasing with a fluctuating tendency. F3 increased sharply in both soils within the first irrigation period (0–1 d), indicating that more labile fractions were transformed into F3 in a fairly short time (<1 d). During the second drainage period (9–16 d), the proportion of F3 in both soils decreased significantly, indicating that F3 was redistributed in both soils during this period. It can be observed in Figure 3C,D that the content of amorphous Fe oxides decreased rapidly within 9–16 d. By contrast, the content of crystalline Fe oxides increased markedly, indicating that amorphous Fe oxides can be transformed into Fe oxides with better crystallinity. Hence, the result can be attributed to the decrease in the content of amorphous Fe oxides and the increase in the crystallization of Fe oxides in soils. Compared with the L_T_ and L_S_, the proportion of F3 in the L_S_ was higher than that in the L_T_. The reason for this might be that Fe/Mn oxides became more positively charged in the lower pH of L_S_ and reduce their ability to bind Cd [46].

As shown in Figure 4D, the proportion of the F4 in both soils decreased slowly in the first irrigation period (0–1 d) following the addition of Cd to the soils and increased sharply in the following time period (1–24 d); then, F4 decreased markedly in the fourth irrigation period (24–25 d), finally increased sharply in the following time period, indicating the redistribution of F4 in both soils over intermittent irrigation periods. The reason for the decrease in F4 in the first irrigation period might be due to the enhancement of oxidation dissolution of Cd sulfides during the oxidation condition, resulting in the mobilization of Cd [24]. Inversely, the transformation of sulfate to sulfide can reduce the mobility of Cd when the soil is in oxidition conditions. Nevertheless, different from the first irrigation period, the decrease in F4 in the fourth irrigation period can be attributed to the dissolution of OM. Yan et al. [40] reported that OM may be anaerobically degraded through microbial activity under flooding conditions. The proportion of F3 in the L_T_ and L_S_ reached 16.6% and 11.3% after 32 days; compared to its content at the initial moment (0 d), this represented an increase of 14.0% and 8.7%, respectively. Overall the proportion of F4 in the L_T_ was higher than that in the L_S_ within the whole incubation time, indicating that shallow soils would accelerate the formation of F4 compared to deep soils. 

F5 is the most stable fraction, existing in the crystal structure of primary and secondary minerals [30], and it is generally difficult to release from soils and has low environmental toxicity to be passively absorbed by plants [53]. In this study, the proportion of F5 in both soils maintained an extremely low level (<2.0%) within the whole incubation time; this indicated that more mobile fractions of Cd will not be rapidly transformed into F5 after the addition of exogenous Cd in a short period. The result was consistent with [49], who reported that the Cd contents of F5 remained relatively constant in both deionized water and simulated acid mine drainage treatment soil during flooding due to the low mobility of aluminum-silicate minerals.

### 3.4. Influences of Intermittent Irrigation on Cd Stabilization Processes

**Figure 5:**
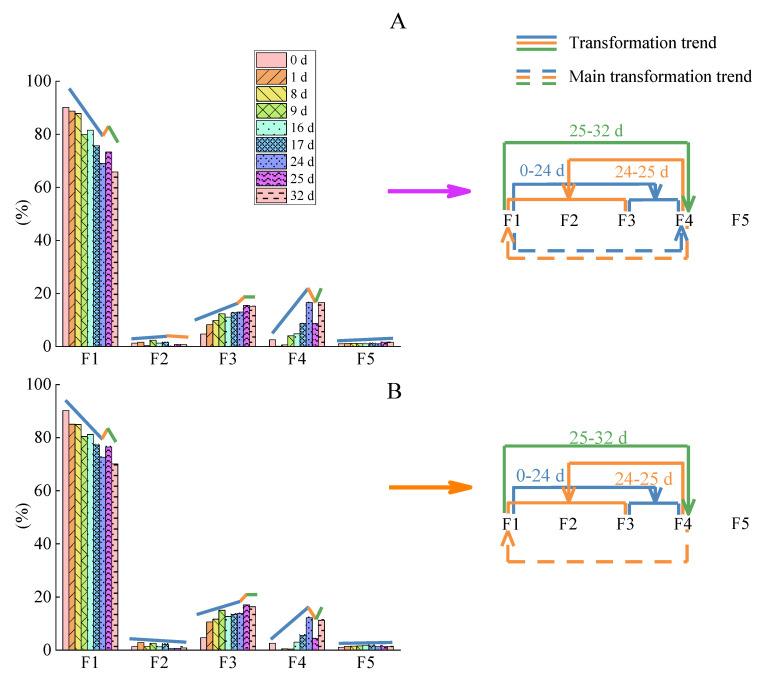
The aging processes of Cd in L_T_ (**A**) and L_S_ (**B**).

As indicated in Figure 5, the aging processes of Cd in both L_T_ and L_S_ soils were divided into three different stages during intermittent irrigation with acid water, and the aging processes continued until the end of the incubation time. As mentioned in 3.3, the proportion of F2 and F5 in both soils maintained a negligible level (<3%) and had no obvious variation trend, indicating that F2 and F5 had little effect on the aging processes. The first stage was from 0 to 24 d, which can be characterized by the decrease in F1 and the increase in F3 and F4, indicating that F1 transformed into F3 and F4. Therefore, the transformation of Cd fractionation was co-controlled by micropore diffusion and the occlusion within organic matter and Fe/Mn hydroxides [45]. Tang et al. [46] reported that when soluble Cd is rapidly adsorbed by the soil, a secondary shift occurred that meant Cd moved from the outer sphere of soil minerals into the inner sphere by the diffusion of micropores. It is worth noting that the transformations of Cd fractions in L_T_ and L_S_ were slightly different in this stage. Different from L_S_, the main transformation during the first stage in L_T_ was from F1 to F4 due to their larger variation. Additionally, compared to the L_S_, the F1 decreased sharply in the L_T_ with the reduction of 20.9%, indicating that the topsoil could promote more labile Cd transformed into immobilizable Cd. 

The second stage in both soils was in the fourth irrigation period (from 24 d to 25 d); F4 decreased, F1 and F3 increased, and the main transformation was from F4 to F1 due to their larger variation. Hence, the aging mechanisms in this stage may be the decomposition of organic matter (F4 decreased), adsorption onto the surface of soil minerals (F1 increased) and resorption and occlusion within Fe/Mn hydroxides (F3 increased) [42]. 

The third stage in both soils was from 25 d to 32 d; F1 decreased and F4 increased without significant changes in other fractions, indicating that the transformation of Cd fractions was from F1 to F4. The transformation of this stage was possibly controlled by intraparticle surface diffusion within micropores and occlusion within organic matter [46]. 

## 4. Conclusions

In this study, soil column leaching experiments were used to simulate the actual irrigation conditions of paddy to investigate the aging processes of exogenous Cd in paddy soils at different depths around mine areas under acid water intermittent irrigation. Obvious changes in soil properties (such as pH, ORP, Fe oxides and DOC) in soils over time under intermittent irrigation were found. Within the whole incubation time, the Cd fractionation in different depths of soils presented a similar change, which can be characterized by the decrease in labile fractions (F1) and the increase in immobilizable fractions (F3 and F4) over time. F3 and F4 were redistributed in both soils over time. Compared to the topsoil, the subsoil had higher bioaccessibility of Cd after short aging processes. 

The Cd aging processes in soils at different depths of soils were both divided into three stages, and the aging processes did not end within 32 days. The main aging processes can be described as more labile fractions transforming into more immobilizable fractions, excluding F4 transforming into F1 and F3 during 24–25 d. The corresponding mechanisms for the transformation of Cd fractions in both soils were micropore diffusion and occlusion within organic matter and Fe/Mn oxides. This study reveals the migration and transformation patterns of Cd fractions and the aging mechanism of Cd in paddy soils under intermittent irrigation with acid water. The results of the study can provide a scientific reference basis for the treatment and remediation of Cd pollution in soils around coal mining areas.

## Figures and Tables

**Table 1:** The physicochemical properties of the soils in this study.

Items	Content	Unit	Items	Content	Unit
pH	5.47		Amorphous Fe oxides	0.51	(mg/kg)
Clay	10.91	(%)	Total Cd	0.15	(mg/kg)
Silt	69.16	(%)	Exchangeable Cd	0.012	(mg/kg)
Sand	19.93	(%)	Carbonates bound Cd	0.014	(mg/kg)
Specific area	0.74	(m^2^/g)	Fe and Mn oxides bound Cd	0.031	(mg/kg)
DOC	25.39	(mg/L)	Organic matter and secondary sulfide bound Cd	0.075	(mg/kg)
Free Fe oxides	18.45	(mg/kg)	Residual Cd	0.022	(mg/kg)

**Table 2:** The modified Tessier’s sequential extraction procedure.

Extraction Steps	Extracted Fractionation	Extracting Solution	Extraction Conditions
1	F1	40 mL 1 mol/L MgCl_2_	Shake 4 h, room temperature
2	F2	40 mL 1 mol/L NaOAc	Shake 16 h, room temperature
3	F3	40 mL 0.25 mol/L NH_2_OH·HCl	Shake 22 h, room temperature
4	F4	15 mL 30% H_2_O_2_ +9 mL HNO_3_	Shake 2 h, 83 °C ± 2 °C in the water bath
		9 mL 30% H_2_O_2_	Shake 2 h, 83 °C ± 2 °C in the water bath
		7.5 mL 9.6 mol/L NH_4_OAc + 20% HNO_3_	Shake 30 min, room temperature
5	F5	aqua regia	Digestion, 240 °C

## Data Availability

The datasets generated and/or analyzed during the current study are not publicly available.
